# Blood Transfusion Reactions in Elderly Patients Hospitalized in a Multilevel Geriatric Hospital

**DOI:** 10.1155/2014/178298

**Published:** 2014-04-03

**Authors:** E. Lubart, R. Segal, N. Tryhub, E. Sigler, A. Leibovitz

**Affiliations:** ^1^Geriatric Department, Shmuel Harofeh Medical Center, Affiliated with the Sackler Faculty of Medicine, Tel Aviv University, 75300 Beer Yaakov, Israel; ^2^Hematologic Disease Unit, Kaplan Medical Center, 76470 Rehovot, Israel

## Abstract

*Background/Objectives*. Blood transfusion is a critical issue for patients with chronic diseases such as heart failure, chronic kidney disease, and malignancy. However, side effects are not rare. The purpose of the study is to evaluate the frequency of adverse blood transfusion reactions in hospitalized elderly patients during a one-year period. *Design/Setting/Participants*. Blood transfusion reactions such as fever, chills, dyspnea, and others following blood transfusions in hospitalized geriatric patients during one-year period were examined. *Results*. 382 blood units (242 patients) were administered during the study period. In 40 (11%) cases, blood transfusion reactions occurred. Fever was the most common reaction in 29 cases (72%), four (10%) had shortness of breath, and 3 (8%) had vomiting and chills each. There were no lethal cases in the 24-hour period following blood transfusions. *Conclusion*. A relatively low rate of adverse blood transfusion reactions occurred in our geriatric patients. We may speculate that this is related to underreporting of minor symptoms due to the high percentage of demented patients in this population.

## 1. Introduction

A recent US study found increasing demand for blood transfusion associated with the increased aging of the population [1]. Blood transfusion is an important supportive treatment of cancer patients most of whom are anemic [2], and it is a critical issue for patients with chronic diseases such as heart failure and chronic kidney disease [3, 4]. Anemia should be considered as a factor that reduces quality of life and a risk for early death [5]. The aim of anemia management should be to restore patient functionality and quality of life by restoring effective red cell volume [6]. One of the considerations for decision of blood transfusion is the possibility of adverse reactions [7–10]. The most common complication of transfusion is febrile and chill-rigor reactions, nonhemolytic and the most serious complications are acute hemolytic reaction due to ABO incompatibility [11]. Febrile nonhemolytic transfusion reaction (FTHTR) is an acute (<24 hours) immunological transfusion reaction [12]. Early recognition of symptoms suggestive for a transfusion reaction and prompt reporting to the blood bank are essential.

In view of the increasing needs of blood transfusion in geriatric patients, we decided to examine all adverse blood transfusion reactions in hospitalized patients during one-year period.

## 2. Methods

The study was performed in a Tel Aviv university affiliated 400-bed multilevel geriatric hospital. Patients are admitted from the community, nursing homes, and general hospitals to the acute, rehabilitation, or long-term care wards. All the patients who received packed red blood cells transfusion during the study year were included in this study. Data was collected retrospectively from the records of the charts of patients. We defined blood transfusion reactions such as objective symptoms, recorded by staff, fever, chills, shortness of breath, and vomiting within 24 hours after packed red blood cells transfusion was administrated. Fever was defined as unexpected temperature rise (≥38° or ≥1°C above baseline, if baseline ≥ 37°) during or shortly after transfusion [12]. Vital signs such as blood pressure, pulse, peripheral oxygen saturation, and breaths number were measured five minutes after beginning of blood transfusion and every 15 minutes during first hour thereafter according to guidance of blood transfusion.

The study was approved by the local ethical committee.

The clinical and laboratory data were coded and analyzed by using the SPSS software (SPSS 14). The determination of frequencies was performed by chi square test.

## 3. Results

Two hundred forty-two patients received 382 blood units during the study year (2008). Relevant demographic and clinical data are presented in Table 1. Most patients were female; the median age was 82 ± 9; fifty percent were hospitalized in acute geriatric departments, and 48% of them were demented and 45% bed ridden.

151 patients (63%) received only one unit of packed cells and 67 patients (28%) two units. The causes of anemia were as follows: the most common type was anemia of chronic disease (63%) followed by iron deficiency anemia (19%), folic acid (17%), and vitamin B 12 (9%) deficiency. Decision for blood transfusion was related to the hemodynamic state of the patients that although treated with ferrum, folate, or vitamin B 12 according to the kind of their deficiency, remained anemic and clinically necessitated it. The data on the transfusion reactions is presented in Table 2. In 40 (11%) cases, blood transfusion reactions occurred. Fever was the most common reaction; 29 (72%) of those 40 cases were with reactions. Chills and vomiting were observed in three cases each (8%); four (10%) had shortness of breath. There were no lethal cases in the first 24 hours following blood transfusions. In all cases of febrile reaction, the blood sample was examined for contamination and no cases of pathogenic bacteria were identified. Neither cases of ABO or Rh-factor incompatibility were detected in this study.

## 4. Discussion

During one year in our multilevel geriatric hospital, 382 packed red blood cells transfusion were performed. Blood transfusion reactions occurred in 40 (11%) cases within 24 hours after transfusion. Fever was the most common of all side effects: 29 (72%). We were surprised of the relatively low rate of adverse blood transfusion reactions in this geriatric debilitated population.

Menis et al.'s study [13] shows potential usefulness of databases in assessment of blood utilization, transfusion-related complication, and risk factors among United States' elderly in the outpatient setting. Our study is the first one that surveys blood transfusions reactions in a geriatric inpatient setting. Therefore, comparing data was performed with that of general hospitals and it shows comparable incidence of blood transfusion reactions [14–16]. An overall incidence of transfusions reactions of 8.7% was reported, with febrile nonhemolytic transfusion reaction in 65% of these [14]. Another study by Py et al. reports that febrile reactions (47%) are the main reaction in their study on 100 general hospitals [15]. Narvios et al. [16] reported minor adverse reactions including low grade fever in 19% cases and itching in 41%. Authors concluded that underreporting of minor transfusion reactions exists. They suggest that careful evaluation of any suspected event of transfusion reaction should be referred to the transfusion medicine physicians of blood bank who will evaluate each case and discuss it promptly with the attending physician.

Most patients hospitalized in our multilevel geriatric medical center are frail elderly with a high rate of comorbidities, immobilization, and cognitive impairment. Since many such patients are noncooperative, they do not report minor reactions such as itching, nausea, dizziness, and others. Therefore, noncomplaining initial minor symptoms related to adverse blood transfusion reactions may endanger these patients. Hypotension can be a manifestation of several transfusion reactions: acute hemolytic, bacterial contamination, transfusion-related acute lung injury, and anaphylaxis. In the rare cases, hypotension is the only manifestation of a transfusion reaction [17, 18]. We suppose that this sign may be silent and not recognized in demented patients such as our study population.

The main limitation of this study is its being a retrospective analysis of data of only one year. For better detection and understanding of the reactions associated with blood transfusion in elderly debilitated inpatients, a prospective study should be performed and compared with results from other geriatric hospitals.

## Figures and Tables

**Table 1:** Demographic and clinical data (242 elderly blood transfusion recipients).

Age	
(year ± SD)	82 ± 9
Gender	
Female	143 (59)
Male	99 (41)
Department	
Acute	127 (52)
Skilled nursing	80 (33)
Rehabilitation	35 (15)
Comorbidities diseases	*N* (%)
Hypertension	146 (60)
Dementia	117 (48)
Diabetes mellitus	93 (38)
Chronic renal failure	86 (36)
Decubitus ulcer	84 (35)
CVA*	73 (30)
CHF**	73 (30)
Drugs	*N* (%)
Beta-blockers	97 (40)
Laxatives	97 (40)
Diuretics	85 (35)
Proton pump inhibitors	80 (33)
Benzodiazepines	80 (33)
H-2-Blockers	76 (31)
ACE inhibitors	73 (30)

*CVA: cerebrovascular accident.

**CHF: congestive heart failure.

**Table 2:** Blood transfusion's reactions *n* (%) (out of 382 blood units).

	Fever (nonhemolytic)	Shortness of breath	Vomiting	Rash	Chills	Hemolytic	Total
24 hours	29 (8)*	4 (1.04)	3 (0.8)	1 (0.3)	3 (0.8)	—	40 (10.5)

*Percent out of 382 blood units.
